# Entzündliche Ursachen von Schlaganfällen – Diagnostik und Therapie

**DOI:** 10.1007/s00115-024-01711-8

**Published:** 2024-07-30

**Authors:** Antje Schmidt-Pogoda, Frederike A. Straeten, Carolin Beuker, Nils Werring, Jens Minnerup

**Affiliations:** Klinik für Neurologie mit Institut für Translationale Neurologie, Universitätsklinikum Münster, Albert-Schweitzer-Campus 1, Gebäude A1, 48149 Münster, Deutschland

**Keywords:** Primäre Angiitis des zentralen Nervensystems, Sepsis, Riesenzellarteriitis, Meningitiden, Bakterielle Endokarditiden, Primary angiitis of the central nervous system, Sepsis, Giant cell arteritis, Meningitis, Bacterial endocarditis

## Abstract

Entzündliche Schlaganfallursachen sind häufig und stellen oft eine diagnostische und therapeutische Herausforderung dar, da es hierzu kaum randomisierte Studien gibt und für viele Konstellationen keine klaren Leitlinienempfehlungen existieren. Nach Veröffentlichung der Empfehlungen der Europäischen Schlaganfallgesellschaft zur primären Angiitis des zentralen Nervensystems (PACNS) im letzten Jahr hat die Deutsche Gesellschaft für Neurologie (DGN) in diesem Jahr eine sehr klare Leitlinie zur Diagnostik und Therapie der PACNS herausgegeben und die Empfehlungen für die systemischen Vaskulitiden aktualisiert. Häufig treten Schlaganfälle jedoch nicht nur infolge einer primären Gefäßentzündung auf, sondern auch als Komplikation einer anderen Organinfektion. So erleiden ca. 5 % aller PatientInnen mit einer Sepsis, ca. 20 % der PatientInnen mit bakterieller Meningitis und bis zu 40 % der PatientInnen mit bakterieller Endokarditis als Komplikation einen Schlaganfall. Unser Artikel fasst die wichtigsten Charakteristika dieser entzündlichen Ursachen von Schlaganfällen zusammen und fokussiert hierbei insbesondere auch auf aktuelle Empfehlungen zum diagnostischen und therapeutischen Management.

**Zusatzmaterial online:**

Zusätzliche Informationen sind in der Online-Version dieses Artikels (10.1007/s00115-024-01711-8) zwei weitere Tabellen enthalten.

## Abstract (en)

Inflammatory causes of stroke are frequent and often pose diagnostic and therapeutic challenges due to the scarcity of randomized trials and the absence of clear guideline recommendations for many scenarios. Following the publication of the recommendations of the European Stroke Organization on primary angiitis of the central nervous system (PACNS) last year, the German Neurological Society (DGN) has issued very clear guidelines this year on the diagnostics and treatment of PACNS and updated the recommendations for systemic vasculitides; however, stroke often occurs not only as a result of primary vascular inflammation but also as a complication of another organ infection. Approximately 5% of all patients with sepsis, ca. 20% of patients with bacterial meningitis and up to 40% of patients with bacterial endocarditis suffer from a stroke as a complication. This article summarizes the key characteristics of these inflammatory causes of stroke and particularly focuses on the current recommendations for diagnostic and therapeutic management.

## Hintergrund

Der ischämische Schlaganfall ist weltweit eine der häufigsten Todesursachen und häufigste Ursache für eine anhaltende Behinderung im Erwachsenenalter. Die weit überwiegende Anzahl von Schlaganfällen ist Folge vaskulärer Risikofaktoren, insbesondere der arteriellen Hypertonie. Die häufigsten unmittelbaren Ursachen des zerebralen Gefäßverschlusses sind hierbei das Vorhofflimmern sowie arterielle Embolien bzw. lokale Verschlüsse infolge atherosklerotischer Gefäßstenosen. Neben diesen häufigen Schlaganfallursachen, die überwiegend zum Erkrankungsgipfel im höheren Lebensalter beitragen, gibt es eine Vielzahl seltener Schlaganfallursachen mit z. T. einer Häufung bei jüngeren Menschen. In diese Kategorie der seltenen Schlaganfallursachen fallen verschiedenste entzündliche systemische wie auch auf das Zentralnervensystem (ZNS) fokussierte Erkrankungen. Sie sind Gegenstand des vorliegenden Übersichtsartikels. Hierbei liegt der Fokus auf den alltagsrelevanten Aspekten Diagnostik und Therapie, die häufig spezifisch sind.

## Primäre Angiitis des zentralen Nervensystems

Die primäre Angiitis des zentralen Nervensystems (PACNS) ist eine Vaskulitis unbekannter Ursache, die isoliert die Arterien (und weniger häufig die Venen) des Gehirns, des Rückenmarks und der Leptomeningen betrifft [1]. Die geschätzte Inzidenz der PACNS beträgt 2,4 Fälle pro 1.000.000 Personen pro Jahr, dabei sind Männer und Frauen gleich häufig betroffen [2]. In einer rezenten Analyse von 911 PatientInnen mit PACNS lag das durchschnittliche Patientenalter bei Diagnosestellung bei 42 Jahren, die Altersspanne reichte von 24 bis 63 Jahren [3].

Die definitive Diagnose PACNS kann nur bioptisch oder autoptisch gestellt werden

Da die Vaskulitis jegliche Region des ZNS beeinträchtigen kann, variieren die klinischen Erscheinungsformen. Zu den häufigsten Symptomen gehören fokal-neurologische Defizite (63 %), Kopfschmerzen (51 %) und kognitive Beeinträchtigungen (41 %; [3]). Die Kopfschmerzen infolge PACNS werden unterschiedlich beschrieben, neigen jedoch dazu, eher subakut und unterschwellig zu sein, im Unterschied zu den abrupt auftretenden, intensiven Donnerschlagkopfschmerzen, die typisch für das reversible zerebrale Vasokonstriktionssyndrom (RCVS) sind. Schlaganfälle und transiente ischämische Attacken (TIAs) treten oft mehrzeitig und in unterschiedlichen Gefäßterritorien auf [3].

Die Diagnosestellung der PACNS gestaltet sich oft als herausfordernd. Aufgrund der risikobehafteten Therapieoptionen ist jedoch eine große therapeutische Sicherheit gefordert. Die definitive Diagnose kann nur bioptisch oder autoptisch gestellt werden [1]. Sofern die Diagnose nicht bioptisch gesichert werden kann, lässt sich eine wahrscheinliche PACNS diagnostizieren, wenn sowohl angiographische als auch magnetresonanztomographische (MRT-)Untersuchungen typische Merkmale aufweisen und diese durch ein für die PACNS spezifisches Liquorprofil ergänzt werden (s. unten; [1]). Die Entnahme einer leptomeningealen und parenchymatösen Biopsie sollte möglichst aus einem MR-tomographisch oder angiographisch betroffenen Bereich erfolgen. Hierdurch kann die Sensitivität der Biopsie auf bis zu 80 % angehoben werden [4]. Bevorzugte Biopsielokalisation sind nichteloquente Areale der nichtdominanten Hemisphäre. Bei positivem Befund finden sich drei unterschiedliche histologische Muster: granulomatös mit multinukleären Zellen (58 %), lymphozytär (28 %) oder nekrotisierend (14 %; [5]). Neben der Diagnosesicherung dient die Biopsie außerdem dem Ausschluss von Differenzialdiagnosen, wie primären ZNS-Lymphomen, Tumoren u. a.

Die konventionelle zerebrale digitale Subtraktionsangiographie (DSA) zeigt bei der PACNS mit Befall mittelgroßer bis großer Gefäße (Medium-to-large-vessel-Variante [MV/LV-PACNS]) typischerweise multilokuläre segmentale Stenosierungen [3]. Diese sind zwar charakteristisch, ausgeprägte arteriosklerotische Veränderungen sowie das reversible zerebrale Vasokonstriktionssyndrom können jedoch ein ähnliches Muster zeigen. Bei der Small-vessel-Variante (SV-PACNS) bleibt die DSA unauffällig [6]. In diesen Fällen ist eine histologische Diagnosesicherung durch eine Hirnbiopsie erforderlich.

**Abb. 1:**
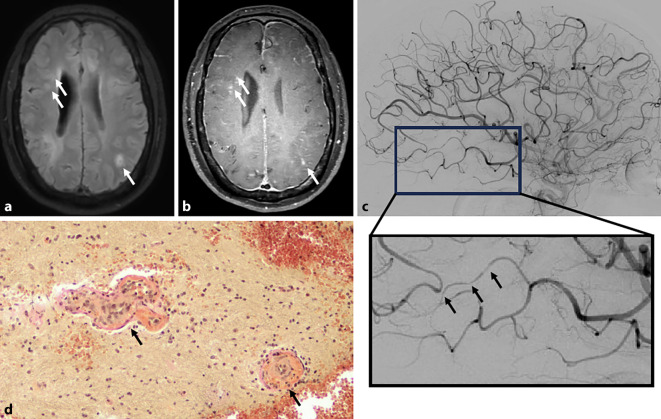
Charakteristische Befunde bei Patienten mit primärer Angiitis des zentralen Nervensystems (PACNS). **a** Magnetresonanztomographie (MRT) mit FLAIR(„fluid attenuated inversion recovery“)-Sequenz, die multiple demyelinisierende Läsionen aufzeigt. **b** MRT unter Verwendung von Black-blood-Sequenzen, welche einerseits eine teilweise deutliche Kontrastmittelaufnahme der Läsionen demonstrieren (*Pfeile*) und andererseits eine ausgeprägte Kontrastmittelanreicherung der Pachy- und Leptomeningen zeigen, als Indikator der floriden Entzündung. **c** Digitale Subtraktionsangiographie (DSA), die multiple Kaliberschwankungen aufweist, exemplarisch im vergrößerten Bildausschnitt durch *Pfeile* markiert. **d** Elastica-van-Gieson(EvG)-Färbung eines Biopsats mit durch *Pfeile* gekennzeichneten Blutgefäßen, die Gefäßwandnekrose und einzelne Lymphozyten in der Gefäßwand zeigen (Vergr.: 200-fach)

Magnetresonanztomographisch zeigen sich typischerweise multifokale Läsionen in der weißen Substanz, gadoliniumaufnehmende Läsionen und ein leptomeningeales Enhancement [6]. Gradientenechosequenzen können petechiale Hämorrhagien darstellen. Das sog. „black-blood imaging“ kann bei der MV/LV-PACNS den klassischen Befund einer konzentrischen, segmentalen Kontrastmittelaufnahme ergeben [7], birgt jedoch das Risiko falsch-positiver Befunde. Wichtig in der Verlaufsdiagnostik der PACNS ist, dass eine Kontrastmittelaufnahme der Gefäßwand trotz Immuntherapie persistieren kann und nur partiell auf Rückfälle hinweist [7]. Abb. 1 zeigt typische MR-tomographische, angiographische und histopathologische Befunde.

Die serologische Labordiagnostik dient dem Ausschluss von Differenzialdiagnosen, da serologische Parameter bei der auf das ZNS-begrenzten PACNS in der Regel normwertig sind. Die Liquordiagnostik ist obligat zum Ausschluss einer infektiösen Genese und zeigt bei 65–75 % der Patienten mit PACNS eine moderate lymphomonozytäre Pleozytose und/oder eine Eiweißerhöhung [3]. Bei einer Pleozytose > 250/µl sollte hingegen an andere, v. a. infektiöse Erkrankungen gedacht werden [1]. Insbesondere sollte auch mittels PCR („polymerase chain reaction“) und Antikörperindizies aus Liquor und Blut eine Varizella-Zoster-Virus(VZV)-bedingte Vasopathie ausgeschlossen werden. Diese tritt insbesondere bei Kindern häufig auf [8].

In der Therapie der PACNS wird zwischen Induktions- und Erhaltungstherapie unterschieden. Das Ziel der Induktionstherapie ist die Remission, das Ziel der Erhaltungstherapie der Remissionserhalt und das Verhindern von Rezidiven. Die aktuellen Leitlinien der Deutschen Gesellschaft für Neurologie (DGN) empfehlen eine initiale intravenöse Kortikoidstoßtherapie über 3 bis 5 Tage mit anschließender Oralisierung auf 1 mg/kgKG und nachfolgender schrittweiser Dosisreduktion nach EULAR(European League Against Rheumatism)-Empfehlungen für ANCA-assoziierte Vaskulitiden. Zusätzlich wird eine Induktionstherapie mit Cyclophosphamid für 6 Monate empfohlen [9]. Im Anschluss empfehlen viele ExpertInnen aufgrund des Nebenwirkungsprofils von Cyclophoshamid eine Umstellung auf Azathioprin, Mycophenolat-Mofetil oder Methotrexat [1]. Nach 24-monatiger Rezidivfreiheit unter einer Erhaltungstherapie wird das Erwägen eines Auslassversuchs empfohlen [9].

## Riesenzellarteriitis und andere systemische Vaskulitiden mit ZNS-Beteiligung

Die Riesenzellarteriitis (RZA) ist in Europa die häufigste idiopathische Vaskulitis und tritt ab einem Alter von 50 Jahren mit zunehmender Häufigkeit auf (Peak in der 7. Lebensdekade; [10]). Das Lebenszeitrisiko beträgt für Frauen ca. 1 % und für Männer ca. 0,5 % [10]. Als Großgefäßvaskulitis befällt die Riesenzellarteriitis insbesondere die Aorta und ihre direkten Abgänge [10]. Klinisch typisch ist die Manifestation mit neuartigen, akut-subakut einsetzenden, häufig temporal lokalisierten Kopfschmerzen, die meist nicht analgetikaresponsiv sind [11]. Ein pathognomonisches, aber nicht obligates Symptom ist die Kiefer‑/Kau-Claudicatio infolge einer Beteiligung der die Massetermuskulatur versorgenden Äste der Arteria carotis externa [11]. Weitere typische Symptome einer RZA sind Sehstörungen wie Doppelbilder oder (transienter) Visusverlust infolge einer arteriitischen anterioren ischämischen Optikusneuropathie (AION), eine Berührungsempfindlichkeit der Kopfhaut (Kämmschmerzen) und konstitutionelle Allgemeinsymptome wie Gewichtsverlust, Nachtschweiß und Abnahme der körperlichen Leistungsfähigkeit [11].

Schlaganfälle sind eine oft unterschätzte Folge einer RZA-Manifestation

Schlaganfälle stellen eine klinisch bedeutsame und in ihrer Häufigkeit oft unterschätzte RZA-Manifestation (1,5–7 %) dar [6, 12]. Hierbei ist der Befall des vertebrobasilären Stromgebiets charakteristisch [13]. Insbesondere betroffen ist die A. vertebralis proximal des Abgangs der A. cerebelli posterior inferior (Abschnitte V2, V3, proximale V4) mit einem fließenden Übergang von entzündlich-stenotischem zu physiologischem Gefäßlumen („Slope-Zeichen“; [13, 14]).

In der klinischen Untersuchung sollte auf eine Druckdolenz, Verhärtung, Schwellung oder eine abgeschwächte Pulsatilität der Arteria temporalis unter Palpation geachtet werden [11]. Laborchemisch sollten das C‑reaktive Protein (CRP) und die Blutsenkungsgeschwindigkeit (BSG oft erhöht mit Werten bis 100 mm in der ersten Stunde, aber auch Werte < 50 mm möglich) bestimmt werden [11]. Normwerte sind gerade bei vorwiegend intrakraniellem Befall möglich und schließen eine RZA nicht aus [11, 13]. Die primär einzusetzende Bildgebungsmethode zur Darstellung entzündlicher Veränderungen der Gefäßwand (oft mit diskontinuierlichem Befall, „skip lesions“) ist die farbkodierte Duplexsonographie der temporalen und axillären Arterien mittels Linearschallköpfen und einer Frequenz von ≥ 15, besser ≥ 18 MHz für die Temporalarterien [15]. Das „Halo-Zeichen“ der Temporalarterie in Form eines zirkulären echoarmen Wandödems gilt als Leitbefund mit einer hohen Spezifität (96 %; [15]). Eine Biopsie der Temporalarterie sollte Fällen, in denen die Diagnose trotz hohen klinischen Verdachts nicht allein durch die Bildgebung gesichert werden kann, vorbehalten bleiben [9]. Zu beachten ist, dass die Sensitivität beider Untersuchungen unter Initiierung einer Glukokortikoid(GC)-Therapie innerhalb der ersten Tage stark abnimmt [16]. Dennoch darf die GC-Therapie aufgrund oben geschilderter Komplikationen keinesfalls verzögert werden. Die 18-Fluoro-2-Desoxy-D-Glukose-Positronenemissionstomographie-Computertomographie (18F-FDG-PET-CT) ist geeignet zum Nachweis extrakranieller Gefäßbeteiligungen der RZA und ist in Sensitivität und Spezifität der magnetresonanztomographischen Diagnostik deutlich überlegen [15]. Allgemein konsentierte Diagnosekriterien oder ein trennscharfer Biomarker existieren für die RZA nicht. Die Klassifikationskriterien der American College of Rheumatology (ACR)/European League Against Rheumatism (EULAR) von 2022 wurden für wissenschaftliche Zwecke und nicht für die Diagnosestellung im klinischen Alltag validiert.

Bei typischen klinischen Befunden sowie positiver Bildgebung ist die Diagnose RZA sicher zu stellen

Die Diagnose wird auf Basis der klinischen Anamnese und Untersuchung, paraklinischer Befunde (Labor und Bildgebung) und je nach Konstellation mittels additiver histopathologischer Untersuchung gestellt. Bestehen typische klinische Befunde sowie ein positiver Bildgebungsbefund (inklusive Duplexsonographie) ist die Diagnose sicher zu stellen [9]. Ist einer der beiden Befunde isoliert positiv, sollte ein weiteres diagnostisches Verfahren in Form weiterer Bildgebungsmodalität oder histopathologischer Untersuchung ergänzt werden [9]. In aktuellen Studien wird an Vorhersagemodellen gearbeitet, die es ermögliche sollen, mittels klinischer Befunde und Ultraschalldiagnostik die RZA zuverlässig von RZA-Mimics trennen zu können [17].

Entscheidend bei der Therapie der Riesenzellarteriitis ist die Vermeidung jeglicher Zeitverzögerung. Gemäß der ACR-Leitlinie von 2021 besteht die Initialtherapie in der hochdosierten oralen (40–60 mg Prednisolon/Tag) oder im Falle drohender neurologischer (Visus‑)Komplikationen intravenösen (500–1000 mg Methylprednisolon/Tag) GC-Therapie [18]. Da die meisten RZA-PatientInnen ein hohes Risiko für steroidassoziierte Nebenwirkungen aufweisen, sowie aufgrund der meist erforderlichen hohen GC-Dosierungen, sollte eine steroidsparende Therapie in der Regel primär eingesetzt werden. Zugelassen ist hier der Interleukin-6-Rezeptor-Antikörper Tocilizumab (TCZ) in der wöchentlichen subkutanen Gabe von 162 mg. Unter Tocilizumab muss im Falle von Infektionen sowie in der Rezidivdiagnostik beachtet werden, dass es infolge der Inhibition der Akute-Phase-Proteine zu keinem oder nur verzögertem CRP-Anstieg kommt. Ebenfalls wird Methotrexat als steroidsparende Therapie eingesetzt („off-label“; [9]). Nach Stabilisierung der Erkrankung sollte das GC gemäß GiACTA-Schema über 52 Wochen (ohne steroidsparende Therapie) oder über 26 Wochen (mit TCZ) ausgeschlichen werden ([19]; Abdosierungsschema siehe Tabelle e2 im Onlinezusatzmaterial). Unter GC-Dosierungen > 5 mg/Tag sollten eine Osteoporoseprophylaxe mit täglicher Aufnahme von Vitamin D3 (1000 IE/Tag) und Kalzium (1000–1500 mg/Tag) über die Nahrung oder Supplemente erfolgen. Eine routinemäßige Gabe von Statinen, Thrombozytenfunktionshemmern oder einer oralen Antikoagulation wird nicht empfohlen [9, 18].

Neben der Riesenzellarteriitis können auch die anderen systemischen Vaskulitiden mit einer ZNS-Beteiligung einhergehen [20]. Im Vergleich zur Riesenzellarteriitis haben diese eine wesentlich geringere Inzidenz [20]. Tabelle e1 aus dem Onlinezusatzmaterial gibt eine Zusammenfassung der typischen Symptome, der pathophysiologischen Charakteristika und Therapieempfehlungen.

## Sepsis und systemische Infektionen

Infektionen, insbesondere die Sepsis, steigern das Schlaganfallrisiko in einem vergleichsweise kurzen Zeitfenster signifikant [21, 22]. Eine aktuelle Metaanalyse, die Studien mit insgesamt über 120.000 PatientInnen einschließt, zeigt, dass ca. 5 % aller PatientInnen mit Sepsis einen ischämischen Schlaganfall erleiden [23]. Zu den Mechanismen, durch die systemische Infektionen Schlaganfälle verursachen, zählen Embolien durch neu aufgetretenes („symptomatisches“) Vorhofflimmern, hämodynamische Instabilität und das Auftreten infektionsassoziierter Koagulopathien [24].

In einer Analyse von 49.082 PatientInnen mit schwerer Sepsis trat bei 5,9 % neu diagnostiziertes Vorhofflimmern auf [21]. Weiterhin wurde für diese PatientInnen mit sepsisbedingtem, neu aufgetretenem Vorhofflimmern ein fast 4‑fach erhöhtes Schlaganfallrisiko ermittelt [21]. Auch wenn symptomatisches Vorhofflimmern bei kritisch kranken PatientInnen oft vorübergehend ist [25], kann hierdurch bereits ein erhöhtes Embolierisiko bedingt sein, da sich Vorhofthromben schon innerhalb von 2 Tagen nach Beginn des Vorhofflimmerns bilden können [26]. Die Datenlage zur Antikoagulation bei PatientInnen mit neu diagnostiziertem Vorhofflimmern während einer Sepsis zeigt jedoch ein erhöhtes Blutungsrisiko ohne Risikoreduktion für ischämische Schlaganfälle [27, 28], sodass im Allgemeinen keine Antikoagulation für PatientInnen mit Sepsis und neu diagnostiziertem Vorhofflimmern empfohlen wird.

Die Inzidenz einer sepsisassoziierten klinischen oder subklinischen Koagulopathie wurde in vorausgehenden Arbeiten auf über 80 % geschätzt [29]. Besonders häufig ist die disseminierte intravaskuläre Koagulation (DIC). Hierbei werden prokoagulante Substanzen wie der Gewebefaktor (TF) freigesetzt, die zur Aktivierung der Blutgerinnung führen und dadurch die Bildung von Thromben im Mikrogefäßsystem oder in größeren Gefäßen verursachen. Die umfangreiche Bildung von Thromben führt wiederum zum Verbrauch von endogenen Gerinnungsfaktoren und Thrombozyten mit der Folge einer Verbrauchskoagulopathie [30, 31]. Endorganschäden resultieren aus reduzierter Perfusion, Thrombosen und/oder Blutungen [30, 31]. Die Diagnose der DIC wird klinisch und laborchemisch gestellt [31]. Die klinische Verdachtsdiagnose ergibt sich bei Patienten, die generalisiertes Sickerbluten aus mehreren intravenösen Katheterstellen oder andere Blutungszeichen aufweisen oder bei Patienten mit unerklärten Thrombosen und einer zugrunde liegenden Erkrankung, die für eine DIC prädestiniert. Die Diagnose einer akuten DIC gilt als gesichert, wenn laborchemische Anzeichen für eine Thrombozytopenie, ein Verbrauch von Gerinnungsfaktoren (z. B. Verlängerung der Prothrombinzeit [PT] oder der partiellen Thromboplastinzeit [aPTT], ein niedriges Fibrinogen) und eine Fibrinolyse (z. B. erhöhter D‑Dimer-Wert) festgestellt werden, vorausgesetzt, es gibt keine andere Erklärung für diese Befunde [31].

Noch Monate nach Abklingen der Infektion besteht ein erhöhtes Schlaganfallrisiko

Auch wenn das Schlaganfallrisiko während der akuten Infektion am größten ist, besteht auch noch Monate nach Abklingen der Infektion ein erhöhtes Schlaganfallrisiko [32]. In einer nordamerikanischen Analyse von 120.000 PatientInnen, die aufgrund einer Sepsis oder systemischen Infektion behandelt wurden, erlitten 0,5 % innerhalb eines Jahres einen Schlaganfall [22]. Das Vorliegen einer infektionsbedingten Koagulopathie ging in dieser Analyse mit einem 3‑fach erhöhten Risiko für das Auftreten eines ischämischen Schlaganfalls innerhalb eines Jahres einher. Das Risiko für einen hämorrhagischen Schlaganfall war sogar um das 7‑fache erhöht [22].

Die Behandlung der DIC fokussiert auf die Behandlung der zugrunde liegenden Ursache. Die prophylaktische Gabe prohämostatischer oder antikoagulanter Mittel wird nicht empfohlen. Es sollte eine sorgfältige Überwachung auf Blutungs- und thrombotische Komplikationen erfolgen, um diese gegebenenfalls umgehend zu behandeln. Antifibrinolytika und Prothrombinkomplexkonzentrate sind generell kontraindiziert, da sie das Risiko thrombotischer Komplikationen erhöhen können [33, 34]. Die Gabe von rekombinantem humanem Thrombomodulin erbrachte in einer rezenten Studie bei Patienten mit einer sepsisbedingten Koagulopathie keinen Vorteil hinsichtlich Mortalität und Inzidenz schwerer Blutungen [35].

## Meningitiden

**Abb. 2:**
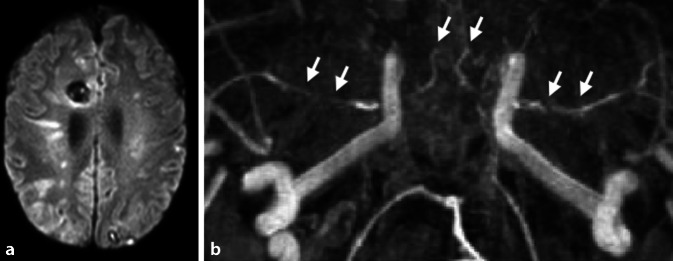
Magnetresonanztomographische Aufnahmen eines Patienten mit bakterieller Meningitis infolge einer Mastoiditis rechts. **a** Die DWI(„diffusion weighted imaging“)-Wichtung zeigt multiple zerebrale Ischämien im vorderen und hinteren Stromgebiet sowie putrides Sediment, hauptsächlich innerhalb der rechten Hemisphäre mit räumlichem Bezug zur Mastoiditis auf dieser Seite. **b** In der TOF(„time-of-flight“)-Angiographie sind multiple Verengungen der intraduralen Abschnitte der hirnversorgenden Gefäße als Ausdruck einer Vaskulopathie nachweisbar (*Pfeile*)

Etwa 20 % der PatientInnen mit bakterieller Meningitis erleiden innerhalb der ersten Wochen nach der Diagnosestellung einen ischämischen Schlaganfall [36]. Neben der bakteriellen Meningitis gehören zu den erregerbedingten Meningitiden auch solche, die durch Viren, Mykobakterien, Pilze oder Protozoen verursacht werden. Infolge der Entzündungsreaktion können bei nahezu allen Formen der Meningitiden sekundär Schlaganfälle auftreten. Die bakterielle Meningitis kann durch das Auftreten typischer klinischer Symptome (Kopfschmerzen, Fieber über 38 °C, Nackensteifigkeit oder Bewusstseinsstörungen) und die Anzahl der Leukozyten im Liquor (über 1000/μl) und/oder den Nachweis bakterieller Mikroorganismen im Liquor diagnostiziert werden [37]. Zerebrovaskuläre Komplikationen, die neben dem ischämischen Schlaganfall auch Hirnvenen- und Sinusthrombosen, Hirnblutungen sowie mykotische Aneurysmen umfassen, tragen zu einem schlechten Outcome der bakteriellen Meningitis bei. Hinsichtlich der zeitlichen Charakteristik kann ein zweigipfeliger Verlauf mit frühen (Tag 3 und 7) und späten (Tag 14) Schlaganfällen beobachtet werden [36]. Der genaue pathophysiologische Mechanismus zur Entstehung von Schlaganfällen im Kontext der bakteriellen Meningitis ist noch nicht vollständig verstanden, jedoch werden der Nachweis von zerebralem Vasospasmus, Vaskulitis, Gerinnungsstörungen, infektiöser Endokarditis und systemischer Entzündungsreaktionen diskutiert [38]. Inwiefern sich die Entstehungsmechanismen früher und später zerebraler Ischämien unterscheiden, ist zum aktuellen Zeitpunkt ungeklärt. Schlaganfälle infolge einer bakteriellen Meningitis treten häufig disseminiert in beiden Hemisphären lokalisiert auf (Abb. 2a). Interessanterweise konnte in einer Fallserie gezeigt werden, dass die Schlaganfälle vor allem in den Versorgungsgebieten von Arterien auftreten, die sich in der Nähe des Infektionsfokus, wie z. B. einer Mastoiditis, befinden [36]. Das Risiko eines ischämischen Schlaganfalls ist unabhängig vom identifizierten Erreger [36]. Eine Vaskulopathie infolge einer bakteriellen Meningitis lässt sich am sensitivsten anhand multipler intraduraler Gefäßverengungen in der Angiographie nachweisen (Abb. 2b).

Spezifische Therapie für Schlaganfälle infolge bakterieller Infektionen der Hirnhäute existiert nicht

Für den klinischen Alltag relevant ist, dass das Auftreten eines Schlaganfalls mit einer höheren Sterblichkeit im Krankenhaus und einem höheren Grad an persistierenden neurologischen Beeinträchtigungen im Vergleich zu PatientInnen ohne Schlaganfall einhergeht [39]. Eine spezifische Therapie für Schlaganfälle infolge bakterieller Infektionen der Hirnhäute existiert nicht. Es wird primär die bakterielle Infektion basierend auf den aktuellen Leitlinienempfehlungen in Form einer empirischen Antibiotikagabe behandelt. Es gibt bislang keine datengestützten Empfehlungen für die akute Behandlung von Schlaganfällen im Zusammenhang mit einer bakteriellen Meningitis. Prinzipiell ist eine mechanische Thrombektomie möglich, von einer intravenösen Lysetherapie sollte in Anbetracht des möglicherweise erhöhten Blutungsrisikos eher abgesehen werden. Es gibt bisher zwar keine gesicherten Daten für den Einsatz von Acetylsalicylsäure (ASS) zur Sekundärprophylaxe nach vaskulitischem Schlaganfall, jedoch würde sich diese Prophylaxe pragmatisch empfehlen.

### Lyme-Borreliose

Weiterhin hervorzuheben sind zerebrovaskuläre Komplikationen infolge einer Borrelieninfektion. Die Lyme-Borreliose ist eine durch Zecken übertragene multisystemische Infektionskrankheit, die in erster Linie durch drei pathogene Spezies der Spirochäten *Borrelia* (*B. burgdorferi, B. afzelii* und *B. garinii*) verursacht wird. Das Nervensystem ist nach der Haut und den Gelenken das mit 10–15 % am dritthäufigsten betroffene Organsystem. Die Lyme-Neuroborreliose präsentiert sich in der Regel als Symptomtrias aus Meningitis, kranialer Neuritis und Radikuloneuritis, kann aber auch komplizierte Verläufe mit Vaskulitis der Hirngefäße aufweisen. Eine assoziierte Vaskulitis wird in der Literatur mit einer Häufigkeit von 0,3–1 % beschrieben und geht mit einem besonders hohen Schlaganfallrisiko einher [40].

Die leitlinienkonforme Behandlung der Lyme-Neuroborreliose umfasst eine 2‑ bis 3‑wöchige Antibiotikatherapie, in der Regel mit intravenösem Ceftriaxon oder oralem Doxycyclin. Die Deutsche Gesellschaft für Neurologie empfiehlt, bei zerebrovaskulären Manifestationen eine additive Behandlung mit Steroiden und/oder Thrombozytenaggregationshemmern in Betracht zu ziehen [40]. Falls die Krankheitsprogression mittels Antibiotika und Steroiden nicht ausreichend kontrolliert werden kann, sollte eine zusätzliche immunsuppressive Therapie mit Cyclophosphamid evaluiert werden [41]. Hinsichtlich der Akutbehandlung eines Schlaganfalls im Kontext einer Lyme-Neuroborreliose gibt es bisher keine systematischen Arbeiten. In der Literatur sind Einzelfälle zur endovaskulären Behandlung eines vaskulitischen Gefäßverschlusses bei Lyme-Neuroborreliose beschrieben [42].

## Bakterielle Endokarditiden

Die bakterielle Endokarditis bezeichnet eine bakterielle Infektion der endokardialen Herzoberfläche, vor allem der Aorten- und Mitralklappe. Ihre Inzidenz ist in den letzten 30 Jahren weltweit von etwa 9,9 auf etwa 13,8 pro 100.000 Einwohner angestiegen. Gleichzeitig hat auch die Mortalität zugenommen [43]. Erst 2023 sind die Duke-Kriterien zur verbesserten Diagnosestellung revidiert worden und beinhalten seitdem weitere diagnostische Mittel wie die Herz-CT und die intraoperative Inspektion der Herzklappen [44]. Zur Diagnosestellung bedarf es entweder zweier Hauptkriterien (mikrobiologische Kriterien, bildgebende Kriterien, intraoperative Klappeninspektion) oder eines Hauptkriteriums mit drei Nebenkriterien (vaskuläre Phänomene, immunologische Phänomene, Fieber, Prädisposition, körperliche Untersuchung oder ein mikrobiologischer oder bildgebender Nachweis, der kein Hauptkriterium erfüllt) oder der Erfüllung von fünf Nebenkriterien [44].

Der Schlaganfall kann das erste Symptom einer Endokarditis sein

Neben direkten Schädigungen des Herzens und Embolien in peripheren Organe (z. B. Niere, Milz) sind ischämische sowie hämorrhagische Schlaganfälle bei bis zu 15–40 % der PatientInnen eine häufige Komplikation der bakteriellen Endokarditis [45, 46]. Ursächlich für die Ischämien sind septische Embolisationen, die sekundär hämorrhagisieren können. Primäre Hämorrhagien werden durch infektiöse (historisch auch mykotische) Aneurysmen und lokale Arteriitiden verursacht [47]. Der Schlaganfall kann dabei das erste Symptom der Endokarditis darstellen. Bereits 4 Monate vor der Diagnosestellung und bis zu 5 Monate nach Endokarditisdiagnose ist das Schlaganfallrisiko relevant erhöht [48]. Gleichzeitig verschlechtert das Auftreten neurologischer Komplikationen das Outcome der Endokarditis [44].

Risikofaktoren sowohl für Ischämien als auch für Hämorrhagien sind vor allem Infektionen durch *Staphylococcus aureus* als auch die Größe der Klappenvegetationen [49]. Bereits ab 10 mm besteht ein relevantes Embolierisiko, bei einer Größe über 30 mm ist, im Vergleich zu kleineren Vegetationen, das Risiko für ischämische Infarkte etwa 2‑fach und für hämorrhagische Schlaganfälle 3,5-fach erhöht. Mitralklappenendokarditiden haben ein höheres Schlaganfallrisiko als solche der Aortenklappe [49]. Beim Auftreten eines ischämischen Schlaganfalles ist die intravenöse Lyse aufgrund des erhöhten Blutungsrisikos kontraindiziert [50]; die mechanische Thrombektomie erscheint jedoch sicher und kann gemäß den bekannten Indikationen angewandt werden [51].

Die bakterielle Endokarditis ist eine der wenigen Schlaganfallätiologien, bei denen keine Thrombozytenfunktionshemmung und/oder Antikoagulation indiziert ist [52]. Die Fortführung einer bereits bestehenden Therapie aufgrund anderer Indikationen mit hohem Thrombembolierisiko, wie z. B. Vorhofflimmern oder hochgradiger extra-/intrakranieller Gefäßstenosen, kann jedoch unter Nutzen-Risiko-Abwägung erwogen werden, sofern keine zerebrale Hämorrhagie besteht [52, 53].

Die Leitlinie der European Society of Cardiology aus dem Jahr 2023 empfiehlt zur Prävention weiterer Embolien die operative Versorgung. Hierbei muss das individuelle Embolierisiko gegen das perioperative Risiko abgewogen werden, da intraoperativ eine Antikoagulation erfolgen muss. Bislang liegen hierzu nur Daten aus Beobachtungs-, nicht aber randomisierten Studien vor. Eine frühe operative Versorgung wird empfohlen, solange keine Einblutung besteht und der/die PatientIn nicht bereits präoperativ komatös ist [52].

Auch bei hämorrhagischen Schlaganfällen scheint eine Verzögerung einer indizierten Operation das Outcome zu verschlechtern [54, 55]. Real-world-Daten zeigen, dass eine indizierte operative Versorgung entgegen den Empfehlungen noch zu selten erfolgt [56].

## Fazit für die Praxis

- Die primäre Angiitis des zentralen Nervensystems (PACNS) ist eine Vaskulitis unbekannter Ursache. In ihrer Folge treten Schlaganfälle und transiente ischämische Attacken oft mehrzeitig und in unterschiedlichen Gefäßterritorien auf.
- Die Riesenzellarteriitis ist eine klinisch bedeutsame und in ihrer Häufigkeit oft unterschätzte Ursache des Schlaganfalls. Klinisch typisch ist die Manifestation mit neuartigen, akut-subakut einsetzenden, häufig temporal lokalisierten Kopfschmerzen, die meist nicht analgetikaresponsiv sind.
- Infektionen, insbesondere die Sepsis, steigern das Schlaganfallrisiko signifikant. Zu den ursächlichen Mechanismen zählen Embolien durch neu aufgetretenes Vorhofflimmern, hämodynamische Instabilität und infektionsassoziierte Koagulopathien.
- Der Mechanismus zur Entstehung von Schlaganfällen im Kontext der bakteriellen Meningitis ist nicht vollständig verstanden. Diskutiert wird der Nachweis von zerebralem Vasospasmus, Vaskulitis, Gerinnungsstörungen, infektiöser Endokarditis und systemischer Entzündungsreaktionen.
- Schlaganfälle sind eine häufige Komplikation einer bakteriellen Endokarditis. Ursächlich für die Ischämien sind septische Embolien, die sekundär hämorrhagisieren können.

## Supplementary Information

Tabelle e1. Zusammenfassung der typischen Symptome, pathophysiologischen Charakteristika und Therapieempfehlungen

Tabelle e2. Abdosierungsschema (GiACTA-Studie)
