# Supplementary material for: Inflammatory causes of stroke—Diagnostics and treatment
Source: Nervenarzt. 2024 Jul 30;95(10):909–19. [Article in German] doi: 10.1007/s00115-024-01711-8 (PMC11427622; doi:10.1007/s00115-024-01711-8)
Supplement: Supplementary file 1 — Tabelle e1. Zusammenfassung der typischen Symptome, pathophysiologischen Charakteristika und Therapieempfehlungen [file 115_2024_1711_MOESM1_ESM.pdf]

**Tabelle 1**

Die primären systemischen Vaskulitiden werden nach ihrer betroffenen Gefäßgröße eingeteilt. Neben der RZA gehört auch die Takayasu-Arteriitis (TAK) zu den Großgefäßvaskulitiden, die Polyarteriitis nodosa (PAN) und die ausschließlich Kinder und Jugendliche betreffende Kawasaki-Erkrankung manifestieren sich in der Regel an mittelgroßen Gefäßen. Die Kleingefäßvaskulitiden werden in jene in Assoziation mit antineutrophilen zytoplasmatischen Antikörpern (*antineutrophil cytoplasmatic antibodies*, ANCA) auftretende und die Immunkomplexvaskulitiden unterschieden. Schließlich gibt es noch die Vaskulitiden, die variable Gefäßgrößen betreffen wie das Behçet- und das Cogan-Syndrom, sowie die Vaskulitiden, die infolge systemischer Erkrankungen wie beispielsweise dem Systemischen Lupus erythematodes auftreten. Vaskulitiden einzelner Organe (wie bspw. die Kutane leukozytoklastische Vaskulitis) sowie die Vaskulitiden wahrscheinlicher Ätiologie (wie bspw. die Tumor-assoziierte Vaskulitis) werden in diesem Artikel nicht behandelt.[1]

Eine Neuerung in der Therapie der ANCA-assoziierten Vaskulitiden (AAV) stellt der orale C5aR-Inhibitor Avocapan dar, der die kumulative Glukokortidkoiddosis bei der GPA und MPA reduziert. Allerdings existieren keine Langzeitdaten für die Avocapan-Gabe (>1 Jahr), sodass eine Langzeitgabe aktuell nicht empfohlen werden kann. Für die Remissionsinduktion und -erhalt der EGPA ohne Organ-gefährdende Symptome steht seit einiger Zeit der IL-5-Antikörper Mepolizumab zur Verfügung.[2]

| Vaskulitis | Gefäßgröße | Alter | typische Symptome | Charakteristika | Therapie |
|---|---|---|---|---|---|
| Riesenzellarteriiris (RZA) | groß | Peak 7. Lebensdekade[3] | neuartige, temporale Kopfschmerzen; Kieferclaudicatio, Sehstörungen/Visusverlust, Berührungsempfindlichkeit der Kopfhaut, konstitutionelle Symptome, Schlaganfälle[4] | Hyperplasie der Gefäßintima infolge der Entzündung, Bildung von granulomatösen „Riesenzellen“[5] | *Remissionsinduktion* mit hochdosierter Glukokortikoidtherapie<br>idealerweise plus direkter Beginn einer steroidsparenden Therapie mit Tocilizumab (TCZ), MTX (off-label)[6] |

| | | | | | |
|---|---|---|---|---|---|
| Takayasu-Arteriitis (TAK)[7] | groß | 2.-3. Lebensdekade | Aorta und ihre Abgangsgefäße, subakuter Beginn, konstitutionelle Symptome, ischämische Schmerzen der Extremitäten, »*pulseless disease*«, renovaskuläre Hypertonie, Angina pectoris/Myokardinfarkte, Carotidynie | granulomatöse Entzündung der Gefäßmedia | GC<br>+<br>MTX, AZA, CYC (cave: junge Frauen), TCZ, Etanercept, Infliximab[7] |
| Polyarteriitis nodosa (PAN)[8] | mittel (kleine Arterien können mit betroffen sein) | meist im mittleren bis höheren Alter (Peak 5.-6. Lebensdekade) | konstitutionelle Symptome, Mononeuritis multiplex, periphere Neuropathie, kardiale Komplikationen, kutane Manifestationen (Noduli, Livedo reticularis), renale Hypertension, abdominale Schmerzen | segmental-transmurale, nekrotisierende Vaskulitis; meist idiopathisch, sekundär infolge Hepatitis B möglich (Abnahme durch Impfung) | GC<br>+<br>AZA, CYC, MTX, MMF<br>+ antivirale Therapie oder PLEX im Falle von HBV[8] |
| Kawasaki-Erkrankung (KD)[9] | mittel – groß | Kinder (meist <5 Jahre) | akute, selbstlimitierende fieberhafte Erkrankung, polymorphes Exanthem (stammbetont), apurulente Konjunktivitis, Stomatitis (»Erdbeerzunge«), Lymphadenopathie | Neutrophilen-mediierte transmurale granulomatös-nekrotisierende Enzündung, Aorta und Koronararterien können mit betroffen sein | IVIG<br>Anakinra, CYC, Infliximab<br>GC<br>Aspirin[9] |
| *ANCA-assoziierte Vaskulitiden (AAV)*[2,10] | | | *allgemeine konstitutionelle Symptome* | *nekrotisierende Vaskulitis,*<br>*ANCA gegen Myeloperoxidase (MPO) oder Proteinase 3 (PR3)* | *Schubtherapie:*<br>GC für 3-5 Tage (iv-Pulsgabe oder p.o. hochdosiert) |

| | | | | | |
|---|---|---|---|---|---|
| Granulomatose mit Polyangiitis (GPA) | klein | Peak 4. Lebensdekade | Destruktionen oberer Respirationstrakt (»Sattelnase«), blutig-borkiger Schnupfen, pulmonale Rundherde, RPGN (»pauci-immun«) | nekrotisierende, granulomatöse Entzündung,<br>cANCA und PR3-AK (70-90 %) | Avacopan (GPA/MPA)[2]<br>*Remissionsinduktion:*<br>Avacopan (GPA/MPA)<br>MTX, AZA, MMF<br>RTX, CYC<br>Mepolizumab (EGPA)<br>*Remissionserhalt:*<br>MTX, AZA, MMF<br>RTX<br>Mepolizumab (EGPA)[10] |
| mikroskopische Polyangiitis (MPA) | klein | Peak 6. Lebensdekade | RPGN, Dyspnoe, Husten (Hämoptysen), alveoläre Blutungen, Mononeuritis multiplex, Purpura der Haut, Arthralgien/Myalgien | Vaskulitis ohne granulomatöse Entzündung, pANCA und MPO-AK (70-90 %) | |
| eosinophile Granulomatose mit Polyangiitis (EGPA)[10] | klein | Erwachsenenalter, im Kindesalter möglich | Asthma, Sinusitis, Polyposis, Eosinophilie, periphere Neuropathie, pulmonale Infiltrate, eosinophile Kardiomyopathie, gastrointestinale Symptome, periphere Neuropathie | eosinophile Gewebsinfiltration mit Vaskulitis;<br>ANCA nur in 30-40 % detektierbar | |
| *Immunkomplexvaskulitiden* | | | häufig Glomerulonephritis | Ablagerung von Immunglobulinen oder Komplement in der Gefäßwand | |
| kryoglobulinämische Vaskulitis (KV)[11,12] | klein | - | v.a. kutane Manifestation (Purpura), membranoproliferative Glomerulonephritis, Mononeuritis multiplex | leukozytoklastische Vaskulitis (kutan), Ausfällen der Kryoglobuline (Ig + Komplement) <37° C; Auslösung durch<br>Hepatitis C, Autoimmunerkrankungen | antivirale Therapie/Therapie der Grunderkrankung,<br>GC, Iloprost, PLEX (Hyperviskositätssyndrom), RTX (schwere Fälle)[11,12] |

| | | | | | |
|---|---|---|---|---|---|
| | | | | (Sjögren-Syndrom, SLE), monoklonale Gammopathie | |
| IgA-Vaskulitis (IGAV)[13] | klein | Kindesalter | kutan limitierte oder systemische Variante möglich (mind. ein weiteres Organ zur kutanen Manifestation; meist Gelenke, GI-Trakt, Niere, selten Lunge oder Nervensystem) | leukozytoklastische Vaskulitis (kutan), IgA-Immunkomplexablagerungen, | meist ambulant möglich, Verlauf in der Regel selbstlimitierend, renale Beteiligung prognoserelevant[13]<br><br>supportive Maßnahmen (Analgesie, Hydration, körperliche Schonung) |
| *Variable Gefäßvaskulitiden* | | | | | |
| Behçet-Syndrom[14] | variabel | v.a. 3.-4. Lebensdekade, nur selten >40 Jahre beobachtet | rezidivierende, schmerzhafte mukokutane Ulzera (oral, urogenital, kutan), okuläre Beteiligung (Uveitis, Hypopyon), Neuro- Behçet, positiver Pathergie-Test | Overlap zwischen autoimmunem und autoinflammatorischem Syndrom, bevorzugter Befall von Venen (endotheliale Aktivierung → Thrombosen), Assoziation mit Haplotyp HLA-B*51 | (geringe Evidenz, Therapie je nach Organmanifestation)<br><br>u.a. GC, Colchizin<br><br>Apremilast (aufgrund Gefäßverengungen),<br><br>AZA, TNFα-Inhibitoren<br><br>OAK unter thrombotischen Komplikationen kontrovers[14] |
| Cogan-Syndrom[15] | variabel | - | Interstitielle Keratitis, Innenohrbeteiligung (Vertigo, Tinnitus, Hörstörungen), selten Aortitis | unklar, Autoantikörper gegen Innenohrantigene | *systemische Vaskulitis:* GC + MTX, AZA, MMF, CYC |

| | | | | | |
|---|---|---|---|---|---|
| | | | | | *lokal:* topische GC[15] |
| Vaskulitiden bei systemischen Erkrankungen (Kollagenosen) | klein | - | Systemischer Lupus erythematodes<br>Sjögren-Erkrankung<br>Mischkollagenosen | antinukleäre Antikörper (ANA) | GC<br>CYC, MMF, AZA, MTX, HCQ<br>RTX, TCZ, BEL (SLE), TNF-α-Inhibitoren[16] |

TAK Takayasu-Arteriitis, GC Glukokortikoide, MTX Methotrexat, AZA Azathioprin, CYC Cyclophosphamid, TCZ Tocilizumab, PAN Polyarteriitis nodosa, MMF Mycophenolat-Mofetil, PLEX Plasmapherese, HBV Hepatitis B-Virus, KD Kawasaki-Erkrankung, IVIG intravenöse Immunglobuline, ANCA anti-Neutrophile zytoplasmatische Antikörper, AAV ANCA-assoziierte Vaskulitiden, MPO Myeloperoxidase, PR3 Proteinase 3, GPA Granulomatose mit Polyangiitis, MPA mikroskopische Polyangiitis, EGPA eosinophile Granulomatose mit Polyangiitis, RPGN rapid-progessive Glomerulonephritis, cANCA zytoplasmatisches Muster, pANCA perinukleäres Muster, RTX Rituximab, KV kryoglobulinämische Vaskulitis, Ig Immunglobulin, IGAV IgA-Vaskulitis, IgA Immunglobulin A, HLA Humanes Leukozytenantigen, TNF Tumornekrosefaktor, OAK orale Antikoagulation, HCQ Hydrocychloroquin, BEL Belimumab, SLE Systemische Lupus erythematodes.


1. Jennette JC, Falk RJ, Bacon PA, et al. 2012 revised International Chapel Hill Consensus Conference Nomenclature of Vasculitides. Arthritis Rheum. 2013;65(1):1-11.
2. Hellmich B, Sanchez-Alamo B, Schirmer JH, et al. EULAR recommendations for the management of ANCA-associated vasculitis: 2022 update. Ann Rheum Dis. 2024;83(1):30-47.
3. Schirmer JH, Aries PM, Balzer K, et al. [S2k guidelines: management of large-vessel vasculitis]. Z Rheumatol. 2020;79(Suppl 3):67-95.
4. van der Geest KSM, Sandovici M, Brouwer E, Mackie SL. Diagnostic Accuracy of Symptoms, Physical Signs, and Laboratory Tests for Giant Cell Arteritis: A Systematic Review and Meta-analysis. JAMA Intern Med. 2020;180(10):1295-1304.
5. Dejaco C, Brouwer E, Mason JC, Buttgereit F, Matteson EL, Dasgupta B. Giant cell arteritis and polymyalgia rheumatica: current challenges and opportunities. Nat Rev Rheumatol. 2017;13(10):578-592.
6. Kraemer, Berlit et al. Zerebrale Vaskulitis und zerebrale Beteiligung bei systemischen Vaskulitiden und rheumatischen Grunderkrankungen, S1-Leitlinie, 2024, in: Deutsche Gesellschaft für Neurologie (Hrsg.), Leitlinien für Diagnostik und Therapie in der Neurologie. Online: www.dgn.org/leitlinien
7. Grayson PC, Ponte C, Suppiah R, et al. 2022 American College of Rheumatology/EULAR classification criteria for Takayasu arteritis. Ann Rheum Dis. 2022;81(12):1654-1660.
8. Chung SA, Gorelik M, Langford CA, et al. 2021 American College of Rheumatology/Vasculitis Foundation Guideline for the Management of Polyarteritis Nodosa. Arthritis Care Res (Hoboken). 2021;73(8):1061-1070.

**9.** Gorelik M, Chung SA, Ardalan K, et al. 2021 American College of Rheumatology/Vasculitis Foundation Guideline for the Management of Kawasaki Disease. Arthritis Rheumatol. 2022;74(4):586-596.
**10.** Chung SA, Langford CA, Maz M, et al. 2021 American College of Rheumatology/Vasculitis Foundation Guideline for the Management of Antineutrophil Cytoplasmic Antibody-Associated Vasculitis. Arthritis Rheumatol. 2021;73(8):1366-1383.
**11.** Specker C, Passens D, Schlaak J. [Cryoglobulinemic vasculitis]. Z Rheumatol. 2022;81(4):300-304.
**12.** Moretti M, Ferro F, Baldini C, Mosca M, Talarico R. Cryoglobulinemic vasculitis: a 2023 update. Curr Opin Rheumatol. 2024;36(1):27-34.
**13.** Xu L, Li Y, Wu X. IgA vasculitis update: Epidemiology, pathogenesis, and biomarkers. Front Immunol. 2022;13:921864.
**14.** Yazici Y, Hatemi G, Bodaghi B, et al. Behcet syndrome. Nat Rev Dis Primers. 2021;7(1):67.
**15.** Kessel A, Vadasz Z, Toubi E. Cogan syndrome--pathogenesis, clinical variants and treatment approaches. Autoimmun Rev. 2014;13(4-5):351-354.
**16.** Moosig F, Holle J. [Collagenosis and vasculitis-what is allowed in treatment?]. Z Rheumatol. 2018;77(7):569-575.
