# Supplementary material for: Inflammatory causes of stroke—Diagnostics and treatment
Source: Nervenarzt. 2024 Jul 30;95(10):909–19. [Article in German] doi: 10.1007/s00115-024-01711-8 (PMC11427622; doi:10.1007/s00115-024-01711-8)
Supplement: Supplementary file 2 — Tabelle e2. Abdosierungsschema (GiACTA-Studie) [file 115_2024_1711_MOESM2_ESM.pdf]

Supplement

Folgend ist ein Beispielreduktionsschema aus der *Giant Cell Arteriitis Actemra* (GiACTA) Studie aufgeführt. Dieses Schema wurde in der Studie für Patienten/Patientinnen angewandt, die initial 60 mg Prednisolon ohne steroidsparende Therapie erhielten und darunter eine vollständige Remission erreichten[1]:

Woche 2	50 mg Prednisolon
Woche 3	40 mg Prednisolon
Woche 4	35 mg Prednisolon
Woche 5	30 mg Prednisolon
Woche 6	25 mg Prednisolon
Woche 7	20 mg Prednisolon
Wochen 8 + 9	17,5 mg Prednisolon
Wochen 10 + 11	15 mg Prednisolon
Woche 12	12,5 mg Prednisolon
Wochen 13-16	10 mg Prednisolon
Wochen 17-20	9 mg Prednisolon
Wochen 21-24	8 mg Prednisolon
Wochen 25-28	7 mg Prednisolon
Wochen 29-32	6 mg Prednisolon
Wochen 33-36	5 mg Prednisolon
Wochen 37-40	4 mg Prednisolon
Wochen 41-44	3 mg Prednisolon
Wochen 45-48	2 mg Prednisolon
Wochen 49-52	1 mg Prednisolon
ab Woche 53	Prednisolon absetzen

Folgendes Schema wurde für Patienten/Patientinnen in der GiACTA-Studie angewandt, die ergänzend zu der Prednisolontherapie (initial 60 mg/Tag) mit Tocilizumab behandelt wurden und hierunter eine vollständige Remission erreichten[1]:

Woche 2	50 mg Prednisolon + 1x 162 mg TCZ sc.
Woche 3	40 mg Prednisolon + 1x 162 mg TCZ sc.
Woche 4	35 mg Prednisolon + 1x 162 mg TCZ sc.
Woche 5	30 mg Prednisolon + 1x 162 mg TCZ sc.
Woche 6	25 mg Prednisolon + 1x 162 mg TCZ sc.
Woche 7	20 mg Prednisolon + 1x 162 mg TCZ sc.
Woche 8	15 mg Prednisolon + 1x 162 mg TCZ sc.
Wochen 9-10	12,5 mg Prednisolon + 1x 162 mg TCZ sc.
Woche 11	10 mg Prednisolon + 1x 162 mg TCZ sc.
Woche 12	9 mg Prednisolon + 1x 162 mg TCZ sc.
Woche 13	8 mg Prednisolon + 1x 162 mg TCZ sc.

Woche 14	7 mg Prednisolon + 1x 162 mg TCZ sc.
Wochen 15+16	6 mg Prednisolon + 1x 162 mg TCZ sc.
Wochen 17+18	5 mg Prednisolon + 1x 162 mg TCZ sc.
Wochen 19+20	4 mg Prednisolon + 1x 162 mg TCZ sc.
Wochen 21+22	3 mg Prednisolon + 1x 162 mg TCZ sc.
Wochen 23+24	2 mg Prednisolon + 1x 162 mg TCZ sc.
Wochen 25+26	1 mg Prednisolon + 1x 162 mg TCZ sc.
ab Woche 27	Prednisolon absetzen

Es muss bedacht werden, dass die GiACTA-Studie nicht darauf ausgelegt war, ein optimales Ausdosierungsschema für Glukokortikoide zu erarbeiten.

1. Stone JH, Tuckwell K, Dimonaco S, et al. Trial of Tocilizumab in Giant-Cell Arteritis. N Engl J Med. 2017;377(4):317-328.
